# Cardiovascular Function and Exercise Capacity in Childhood Cancer Survivors

**DOI:** 10.3390/jcm11030628

**Published:** 2022-01-26

**Authors:** Barbara Reiner, Irene Schmid, Thorsten Schulz, Jan Müller, Alfred Hager, Julia Hock, Peter Ewert, Cordula Wolf, Renate Oberhoffer-Fritz, Jochen Weil

**Affiliations:** 1 Institute of Preventive Pediatrics, Technical University Munich, 80992 Munich, Germany; thorsten.schulz@tum.de (T.S.); j.mueller@tum.de (J.M.); renate.oberhoffer@tum.de (R.O.-F.); 2 Division of Pediatric Hematology and Oncology, Department of Pediatrics, Dr. von Hauner Children’s Hospital, University Hospital Munich, LMU Munich, 80337 Munich, Germany; Irene.Schmid@med.uni-muenchen.de; 3 Department of Pediatric Cardiology and Congenital Heart Disease, German Heart Center Munich, Technical University Munich, 80636 Munich, Germany; hager@dhm.mhn.de (A.H.); hock@dhm.mhn.de (J.H.); ewert@dhm.mhn.de (P.E.); wolf@dhm.mhn.de (C.W.); weil@dhm.mhn.de (J.W.)

**Keywords:** childhood cancer survivors, long-term side effects, cardiovascular dysfunction, anthracyclines, carotid intima-media thickness

## Abstract

Introduction: Childhood cancer survivors (CCS) might be at high risk of additional chronic diseases due to cardiotoxic side effects. The aim of this study was to analyze long-term side effects of cancer therapy on vascular structure/function, cardiac biomarkers and on physical activity. Methods: In total, 68 asymptomatic patients aged 16–30 years with childhood cancer (diagnosed 10.6 ± 3.9 years ago) were examined from 2015–2020. (Central) blood pressure and pulse wave velocity were registered via the oscillometric method, while carotid intima-media thickness (cIMT) was measured non-invasively by ultrasound. cIMT values of patients were compared to healthy controls (*n* = 68; aged 22.3 ± 3.5 years). Patients’ exercise capacity was recorded. The plasma N-terminal pro-brain natriuretic protein (NTproBNP) and troponin levels were measured as cardiac biomarkers. CCS were categorized in groups with low, moderate and high anthracyclines. Results: No differences were found in cIMT between patients and controls as well as between patients with various anthracycline dosage. Patients with high dose anthracyclines showed a significant lower performance versus patients with moderate dose anthracyclines (84.4% of predicted VO_2_peak; *p* = 0.017). A total of 11.6% of CCS had abnormal NTproBNP values which correlated with received anthracycline dosage (*p* = 0.024; r = 0.343). Conclusion: NTproBNP levels and exercise capacity might be early markers for cardiovascular dysfunction in CCS and should be included in a follow-up protocol, while cIMT and troponin seem not to be adequate parameters.

## 1. Introduction

In Germany, around 2183 persons aged below 18 years develop cancer every year. The survival rate has increased to about 82%, which is why long-term effects of illnesses and therapies are becoming more and more important [1].

Childhood cancer survivors (CCS) are known for the increased prevalence of cardiovascular diseases as adults [2,3]. Even 5 years after the diagnosis, the consequences of the original cancer and subsequent treatment may influence the likelihood of death [3]. Nowadays, cardiovascular disease is the leading cause of non-cancer mortality in CSS [4].

The predisposition of CCS for additional risk factors such as obesity, hypertension, dyslipidemia, glucose intolerance and metabolic syndrome [5] are, next to the side effects of cancer therapy [6], good reasons for a long-term, preventive approach in follow-up. For follow-up treatment of (asymptomatic) survivors and to reduce the burden of long-term side effects of diagnosis and therapy, an early detection of cardiovascular diseases is necessary.

To prevent cardiovascular events in later life, it is very important to identify predictors by which the individual risk of cardiotoxicity and cardiovascular events can be estimated. So far, the main focus has been on echocardiographic values (e.g., speckle tracking, tissue doppler imaging) in order to identify subclinical cardiac dysfunctions [7]. In this article, however, the focus is on vascular parameters.

Some studies have already found differences in vascular function and structure in CCS soon after therapy compared to controls [8]. Despite the observed abnormalities, the influence of isolated and combined factors (anthracycline dose, volume of radiation, traditional risk factors) are not well known [9]. Since there is also an increased risk that traditional risk factors for cardiovascular events are present in CCS, e.g., due to an inactive lifestyle, the likelihood of early vascular impairments, such as subclinical atherosclerosis and arterial stiffness, increases further [8,10].

In general, the atherosclerotic process begins early in life with an accumulation of fatty streaks [11,12]. A measurement of carotid intima-media thickness (cIMT) can reveal early changes of the arterial wall structure. The measurement of cIMT is non-invasive, has already been used many times in children and helped to detect high-risk groups with abnormal vascular wall thickness [8,13]. Therefore, it is a good surrogate marker to detect apparently healthy people with increased cardiovascular risk [14].

N-terminal pro-brain natriuretic peptide (NTproBNP) and cardiac troponin are serum biomarkers that can be useful to evaluate cardiotoxicity after cancer therapy [15]. While cardiac troponin (cTnT) is a protein and biomarker of myocardial damage, NTproBNP are natriuretic peptides and might help to detect cardiac stress [15] and early left ventricular dysfunction.

CCS meet the recommended level of physical activity less often than healthy peers do [16,17]. Further, several studies showed that the level of physical activity and fitness is impaired in CCS [18]. Knowing that physical activity is beneficial for well-being and health [18,19], as well as its protective effect on mortality [20], it is very important to evaluate and encourage physical activity. A cardio-pulmonary exercise test (CPET) is one of the best objective methods for determining exercise capacity [21].

The aim of this study was to investigate long-term side effects of cancer therapy in young people. Of special interest were early signs of atherosclerosis in these patients, so we measured cIMT, as a surrogate marker. Further, the blood pressure, pulse wave velocity (PWV), laboratory profile/cardiac biomarkers and exercise capacity of CCS were evaluated.

## 2. Materials and Methods

### 2.1. Study Subjects

In 2015, our institutions started a collaboration for an oncologic cardiologic surveillance service with a fixed clinical protocol for CCS (Supplement File S1). For the current analysis, medical charts were reviewed from all CCS that were examined at the pediatric cardiology outpatient unit at the German Heart Center Munich between March 2015 and September 2020. Finally, 68 patients aged 16–30 years (21.0 ± 3.0 years) with a measurement of the carotid intima-media thickness were enrolled in this retrospective study. Age, sex, height, weight, body-mass-index (BMI) as well as details of cancer diagnosis and therapy were abstracted from medical records (Table 1 and Table 2). The patients belonged to the following diagnostic groups: Acute Lymphoblastic Leukemia, ALL (*n* = 23), ALL relapse + stem cell transplantation (*n* = 8), Hodgkin’s Lymphoma (*n* = 17), Sarcoma (*n* = 15), Others (*n* = 6). On average, they were diagnosed aged 10.4 ± 4.8 years. The average time since their diagnosis was 10.6 ± 3.9 years (min. 3.8 years; max. 19.6 years).

For subgroup comparisons, the patients were classified based on the received anthracycline dose12: (1) less than 100 mg/m^2^; (2) 100–250 mg/m^2^; (3) more than 250 mg/m^2^. 

Because cIMT norm values are not transferrable [22], we recruited our own control group (*n*= 68; 22.3 ± 3.5 years) from the same setting (parts of these data are already published elsewhere [13,23]). Therefore, we used the same material, standardized ultrasound protocol and same sonographers.

For detailed analyses, only patients with data in all additional required parameters were included. Overall, 43 patients were able to perform a cardiopulmonary exercise test and had registered values for age, height, weight, BMI, age at diagnosis, time off therapy, anthracyclines and radiation exposures as well as values for blood pressure (peripheral and central), PWV and laboratory (total cholesterol, low density lipoprotein (LDL) cholesterol, high-density lipoprotein (HDL) cholesterol, amino-terminal pro-brain natriuretic peptide = NTproBNP, high sensitivity troponin T = cTnT).

All patients, controls and their guardians (for children under 18 years of age) gave written consent for anonymous publication of their data. The need for an ethical board evaluation was waived due to the complete retrospective design of the study.

### 2.2. Clinical Protocol

cIMT was measured strictly in accordance with the guidelines of the “Cardiovascular Prevention Working Groups of the Association for European Pediatric Cardiology” [24]. For all cIMT measurements, a semiautomated ultrasound system, the Cardiohealth Station of Panasonic (Yokohama, Japan), was used. The central frequency of the linear-array transducer was 8.9 MHz. In brief, the guidelines state that all patients should be placed in a supine position with their head tilted 45° and the neck slightly stretched. All measurements were performed on the far wall of the common carotid artery and approximately 1 cm distal to the bulb at the end-diastolic phase. In order to obtain meaningful values, the cIMT was measured at four angles (120°, 150°, 210° and 240°) using B-Mode ultrasound. A mean value was calculated from these values for further analyses. Systolic and diastolic blood pressure as well as central blood pressure and PWV were measured after 5 min of rest in a supine position using the oscillometric Mobil-O-Graph® (IEM Healthcare, Stolber, Germany).

Total cholesterol and its subtypes, LDL cholesterol and HDL cholesterol, were analyzed by enzymatic assays and photometric detection on the Cobas C501 analyzer (Roche Diagnostics, Mannheim, Germany). Troponin (cTnT) and NT-proBNP were quantified by electrochemiluminescence immunoassay (ECLIA) methods on the Cobas E411 analyzer (Roche Diagnostics). All analyses were performed as part of routine patient diagnostics in a highly quality-controlled manner in accordance with the guidelines of the German Medical Association (Rili-BaeK) at the Institute of Laboratory Medicine of the German Heart Center. A level of <0.01 ng/mL was defined as normal for cTnT. For NTproBNP we used sex-specific norm values. While values > 170 ng/L are conspicuous for women, values > 100 ng/L are considered abnormal for men.

Exercise capacity was determined on an electronically braked bicycle ergometer (Corival, Lode, Groningen, NL) in an upright position by a symptom-limited cardio-pulmonary exercise test (CPET) with a ramp-wise protocol and breath-by-breath metabolic analysis (Encore, Vyaire Medical, INC, Mettawa, IL, US). The standardized protocol, following the guidelines of the American College of Cardiology and American Heart Association [25], is routine in our institute and often described [26]. All patients who completed their cardiopulmonary exercise test with exhaustion (respiratory exchange ratio > 1.1) were included in further evaluations. For an assessment of the performance, the peak oxygen uptake (VO_2_peak) of each patient is given as a percentage of the individually expected norm [(VO_2_peak (% norm)]. Values < 80% of predicted norm [27,28] were considered as “abnormal”. 

### 2.3. Data Analyses

Continuous variables are presented as mean ± standard deviations, while categorical variables (such as sex) are reported as counts and percentages. For group comparisons, either t-test, Mann–Whitney U Test or Kruskal–Wallis tests for unpaired samples were carried out, depending on the number of groups, sample size and type of distribution. Multiple linear regression analyses adjusted for covariates were used to assess the mean differences in cIMT and values of blood pressure as well as PWV between various groups. Age, sex, height and weight were entered as covariates.

The association of treatment-related parameters and biomarkers were analyzed using Spearman’s rank correlation. Where necessary, the calculations were adjusted for covariates (sex, age, height and weight) in a multiple linear regression model.

For statistical analyses SPSS 23.0 software was used (BMI Corp., Armonk, NY, USA). Two-sided *p*-values < 0.05 were considered statistically significant.

## 3. Results

Patients’ characteristics are shown in Table 1. All of them were in NYHA class I and asymptomatic at the time of follow-up. The mean age at cancer diagnosis was 10.4 ± 4.8 years, and an average of 10.6 ± 3.9 years have passed since then. In our cohort, 64 patients (94.1%) had chemotherapy and 22 of them also received radiotherapy. The mean cumulative anthracycline dose was 261.99 ± 108.76 mg/m².

In a comparison of cIMT between patients and healthy subjects, no differences were detected after adjustment for sex, age, height and weight (*p* = 0.528). Further, there was neither a difference in cIMT between patients with moderate and high anthracyclines (*p* = 0.415) nor between patients with and without radiotherapy (*p* = 0.412) after adjustment for the same covariates. cIMT did not correlate with age at diagnosis (*p* = 0.688) nor with the time duration between diagnosis and follow up (*p* = 0.423). In addition, the patients with different cancer types did not differ in cIMT (*p* = 0.813).

The patient subgroups (moderate, high anthracycline dose) did not differ in age and height, but weight (*p* = 0.012) and BMI (*p* = 0.003). There were no differences in peripheral or central blood pressure, PWV or any other laboratory parameter between the two groups with moderate and high anthracycline dosage (Table 2).

There was no difference in cIMT between patients with normal and abnormal NTproBNP (*p* = 0.983) after adjustment for sex, age, height and weight differences. Further, no correlation of cIMT values to blood pressure or laboratory parameters were detected.

**Figure 1:**
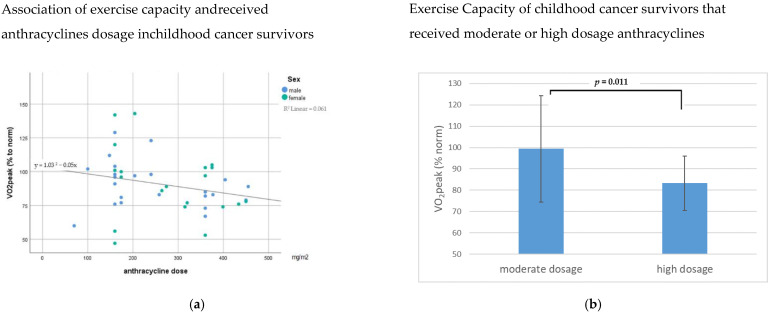
(**a**) Correlation of exercise capacity and anthracycline dosage in childhood cancer survivors about 10 years after cancer diagnosis; VO_2_peak (%to norm) = %predicted peak oxygen uptake; (**b**) Correlation of exercise capacity and anthracycline dosage in childhood cancer survivors about 10 years after cancer diagnosis. Anthracycline dosage (mg/m^2^): moderate = 100–250; high > 250; VO_2_ peak (%to norm) = %predicted peak oxygen uptake.

Only 85.4% of the patients were able to perform a CPET until exhaustion (respiratory exchange ratio (RER) > 1.1). Out of them, 28.6% of the patients had less than 80% of the predicted VO_2_peak. Patients with high anthracyclines performed less than patients with moderate anthracyclines (84.4 ± 10.5 vs. 99.6 ± 24.4% of predicted VO_2_peak.; *p* < 0.017) (Figure 1a,b). A previous radiation treatment showed no additional effect on the performance.

**Figure 2:**
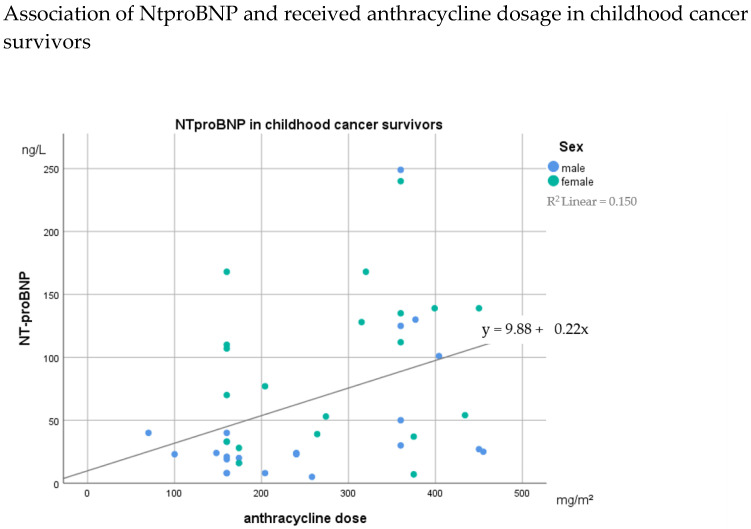
Correlation of N-terminal pro-brain natriuretic peptide (NTproBNP) and anthracycline dosage in childhood cancer survivors after around 10 years.

NTproBNP was abnormal in 11.6% of the patients. A total of 32.6% (14 of 43 patients) had NTproBNP values above 100 ng/L. NTproBNP is positively associated with anthracyclines exposure (r = 0.343; *p* = 0.024) (Figure 2). 

The group with moderate and high anthracyclines did significantly differ in NTproBNP values (42.7 ± 42.5 ng/L vs. 94.91 ± 70.9 ng/L, *p* = 0.005) and this holds true after correction for sex, age, height and weight differences (47.6 ± 13.5 ng/L vs. 90.2 ± 13.2 ng/L; *p* = 0.042). 

Adjusting for the same covariates, there were no differences in RRsys (*p* = 0.237), zRRsys (*p* = 0.579), PWV (*p* = 0.542) or RRdia (*p* = 0.450) between patients with normal vs. abnormal NTproBNP values.

## 4. Discussion

This study showed that despite the fact that all CCS were asymptomatic, a substantial number of patients had a diminished exercise capacity and an elevated NT-proBNP, which correlated with anthracycline dosage. A vascular component (cIMT, central and peripheral blood pressure) of anthracycline toxicity or a continuous cardiotoxic process (cTNT) could not be detected.

The current state of literature does not allow a clear statement about the risk of thickened cIMT in CCS. In our cohort, at a mean time of 10 years after cancer diagnosis, cIMT values do not differ between CCS and healthy controls. This is in accordance with some recently published studies [29,30,31], but at the same time contradicts the results of others [6,32,33].

Li et al. [33] registered increased cIMT values in anthracycline-treated CCS without radiation after an extended follow-up period of approximately 15 years after therapy completion. In long-term survivors of ALL [29,30] or Hodgkin’s Lymphoma [31], no cIMT differences were found comparing patients to healthy controls. Even in patients with high blood pressure, no increased cIMT values were detected [29]. Ociepa et al. concluded that their studied group might be too young and that time since therapy is, with a median of 5.7 years, too short to see damaged arterial walls [29]. Our patients are on average 9 years older, but still show no abnormalities in cIMT. Therefore, either the period is still too short or the known increased risk for these patients is not reflected in increased cIMT values.

The influence of previous radiation therapy is also questionable. For Example, Meeske et al [32] found increased cIMT values in CCS treated with neck irradiation, Zaletel et al. [31] did not. In addition, Sadurska et al. [6] reported increased cIMT in CCS regardless of whether the patients had radiation or not. In contrast to that, Boruwer et al. [34] found no increased cIMT in their large sample of CSS, but the values were significantly increased in the subgroup of CSS with radiotherapy.

The heterogeneity of the study results could be due to the heterogeneity of the examined patient groups (e.g. types of cancer, composition of the sample, mean time from cancer diagnosis), the small number of cases or the differences in the study protocol. On the other hand, the type of treatment (including the dosage of chemotherapy as well as the level and location of radiotherapy) could also have a decisive influence, which was not sufficiently taken into account in every study. For a final assessment, a study with a large number of cases and a cohort of older patients is necessary, which allows comparisons of homogenous groups with the same diagnosis and similar therapy.

None of our patients had cTNT > 0.01. This is in line with Mavinkurve-Groothuis et al. [35], who suspected that this parameter is not usable for detecting late onset cardiotoxicity in CSS. With NTproBNP, however, it is completely different. Abnormal values of NTproBNP were prevalent in 11.6% of CCS, respectively. Studies in the general population have shown that even slightly elevated NTproBNP values (in the upper normal range) are associated with an increased cardiovascular risk and risk of death [36]. In addition, Dixon et al. [37] showed that NTproBNP could be used to identify asymptomatic patients at risk for cardiac events before changes in echocardiography become apparent. At the same time, Mavinkurve-Groothuis et al. [35] suspected that NTproBNP values in asymptomatic patients with normal echocardiography findings could be useful as a predictor for heart failure. In line with our results, however Dixon et al. have shown that there is a close correlation between the received anthracycline dose and later NTproBNP values, which is why they and others called NTproBNP a marker of treatment-related cardiotoxicity [35,37] The sensitivity of NTproBNP as a screening parameter for left ventricular dysfunction was classified as low [38]. Several sources questioned the informative value of this parameter due to poor association with echocardiography findings [37,38]. Especially since there is currently still disagreement about suitable cut-off values [35,38].

On the other hand, numerous studies have already shown an association of anthracycline dose and NTproBNP [35], as well as between increased NTproBNP and cardiac dysfunction in CCS [39,40]. In order to determine the predictive power of NTproBNP values, long-term studies of patients with increased NTproBNP values and at the same time inconspicuous echocardiography are required.

As expected, not all patients (14.6% had RER < 1.1) achieved maximal exhaustion on CPET (e.g., due to physical discomfort or also prosthesis) and were excluded for further subgroup analysis in this field. A total of 28.6% of the remaining CCS showed abnormal performance even years after treatment. It was also demonstrated that CCS with higher anthracycline doses generally performed worse than patients with moderate anthracycline doses—additional radiation had no further effect on exercise performance.

A limited performance of CCS has been shown in various studies [41,42,43]. Further, most studies showed a negative influence of anthracyclines on physical performance [42]. Christiansen et al. [42] showed that a decreased exercise capacity is associated with impaired LV diastolic function and higher anthracycline dose in long-term survivors of childhood ALL. A comparatively inactive lifestyle [44] in CCS could be responsible for the generally limited exercise capacity of these patients in the physical performance test. Fatigue, anxiety, pain and a lack of motivation are some reasons for the sedentary lifestyle [45]. Wherever possible, an active lifestyle should be promoted for all CCS because of the protective effects. However, there is still no consensus on all the mechanisms of cancer therapy with a negative impact on exercise capacity in CCS, and further studies are needed.

## 5. Conclusions

Even in asymptomatic pediatric cancer survivors after anthracycline therapy, NT-proBNP and exercise capacity should be screened to find subtle signs of heart failure. About 10 years after cancer diagnosis, CCS that received high doses of anthracyclines are at a greater risk of high NTproBNP values and reduced exercise capacity than patients with moderate dosage. Long-term observation of patients with high NTproBNP values is necessary to verify the importance of these parameters. Thus, it would be important as the next step to examine a cohort of CCS at about 20 and 30 years after cancer treatment.

Further, the patients’ cIMT and troponin levels were normal. There are no differences between CCS and controls or between the subgroups with moderate and high anthracyclines in these parameters. Consequently, they do not seem to be adequate parameters for the early detection of an increased cardiovascular risk in CCS.

### Limitations

Because most patients had multi-modal treatments (various types of chemotherapy agents or surgeries, radiation exposure, …) it is difficult to address all factors of different cancer and treatment types in order to get homogenous patient groups. For further research, it would help to include a higher number of patients to cluster even more homogenous groups and determine the impact of individual exposures. In addition, a further long-term observation of the study participants would be useful in order to assess the predictive power of preventive markers.

## Figures and Tables

**Table 1:** Patients’ characteristics and details to anthracyclines and carotid intima-media thickness (cIMT).

	N	Sex	Age (years)	Height (cm)	Weight (kg)	BMI	cIMT (mm)
		female *n* (%)	(Mean ± SD)	(Mean ± SD)	(Mean ± SD)	(Mean ± SD)	(Mean ± SD)
CCS total	68	35 (51.5)	21.0 ± 3.1	170.2 ± 10.0	67.5 ± 15.8	23.1 ± 4.1	0.457 ± 0.033
CCS with high anthracyclines	37	21 (56.8)	20.6 ± 2.5	168.6 ± 11.2	62.1 ± 12.9 *1	21.7 ± 3.0 *2	0.454 ± 0.032
CCS with moderate anthracyclines	27	12 (44.4)	21.5 ± 3.8	172.2 ± 7.7	73.6 ± 17.7 *1	24.7 ± 4.7 *2	0.461 ± 0.037
CCS with none/low anthracyclines	4	2 (50.0)	21.1 ± 2.5	171.0 ± 12.9	74.3 ± 9.7	25.6 ± 4.0	0.468 ± 0.015
CCS subgroup with all data	43	20 (46.5)	21.2 ± 3.2	170.8 ± 9.8	68.6 ± 17.6	23.3 ± 4.4	0.459 ± 0.037
Healthy controls	68	35 (51.5%)	22.3 ± 3.5	174.3 ± 9.3	69.0 ± 11.3	22.6 ± 2.6	0.465 ±0.039

Body-mass-index = BMI; numbers = *n*, standard deviation = SD, childhood cancer survivors = CCS; Anthracycline dose (mg/m^2^): low < 100; moderate = 100−250; high > 250; comparison of patients with moderate and high dose of anthracycline *1 *p* = 0.012; *2 *p* = 0.003.

**Table 2:** Data of all childhood cancer survivors with cardiopulmonary exercise test and all other data of patients with low, moderate and high anthracyclines.

	Total	Low/None Anthracyclines	Moderate Anthracyclines	High Anthracyclines	*p*-Value Moderate vs. High Anthracyclines *
	43	*n* = 2	*n* = 20	*n* = 21	
Systolic Blood Pressure (mmHg)	118.6 ± 11.6	122.5 ± 9.2	120.2 ± 11.0	116.7 ± 12.4	0.368
Diastolic Blood pressure (mmHg)	70.0 ± 8.3	69.0 ± 14.1	71.0 ± 9.5	69.1 ± 6.9	0.522
Central systolic blood pressure (mmHg)	108.8 ± 11.7	113.5 ± 9.2	110.0 ± 13.5	107.2 ± 10.3	0.685
PWV	5.0 ± 0.5	5.2 ± 0.6	5.1 ± 0.6	4.9 ± 0.5	0.348
NTproBNP	67.7 ± 62.8	31.5 ± 12.0	42.7 ± 42.5	94.9 ± 70.9	0.005 **
Troponin (ng/mL)	0.005 ± 0.002	0.006 ± 0.003	0.005 ± 0.002	0.005 ± 0.002	0.979
% of predicted VO_2peak_ (*n* = 16 vs. *n* = 17)	90.7 ± 21.2	81.0 ±29.7	99.4 ± 25.0	83.3 ±12.7	0.011 **
cIMT (mm)	0.459 ± 0.037	0.479 ± 0.014	0.460 ± 0.039	0.457 ± 0.037	0.639

* comparing patients with moderate and high anthracyclines with a Mann–Whitney U Test. ** *p*-values < 0.05 are considered statistically significant and are marked in bold. Pulse Wave Velocity = PWV; N-terminal pro-brain natriuretic peptide = NTproBNP; peak oxygen uptake = VO_2peak_; Carotid Intima Media Thickness = cIMT; Anthracycline dose (mg/m2): low < 100; moderate = 100–250; high > 250.

## Data Availability

The data of this study are available from the corresponding author on reasonable request.

## Supplementary Materials

The following are available online at https://www.mdpi.com/article/10.3390/jcm11030628/s1, File S1: Clinical protocol for Childhood Cancer Survivors at the German Heart Centre Munich.
