# Supplementary material for: Cardiovascular Function and Exercise Capacity in Childhood Cancer Survivors
Source: J Clin Med. 2022 Jan 26;11(3):628. doi: 10.3390/jcm11030628 (PMC8837025; doi:10.3390/jcm11030628)
Supplement: Supplementary file 1 [file jcm-11-00628-s001.zip › jcm-1512374-supplementary.pdf]

23.01.2015

Clinical protocol for Childhood Cancer Survivors at the German Heart Centre Munich

Parameters:

- Standardized medical history supported by questionnaires on quality of life and physical activity
- Anthropometrics
- Electrocardiogram (ECG)
- Clinical examination
- Echocardiography (Systolic and diastolic function; Global longitudinal strain)
- Cardio-pulmonary exercise test (CPET) (*previous resting spirometry*)
- Carotid intima-media thickness measurement (cIMT)
- Peripheral blood pressure, Central blood pressure, Pulse wave velocity
- Health-related physical fitness tests *(sport motor performance tests: curl-ups, push-ups, shoulder stretch, trunk lift, sit and reach test)*
- Laboratory profile/cardiac biomarkers
  (including: hemoglobin, erythrocytes, hematocrit, mean corpuscular/cell volume MCV, mean corpuscular hemoglobin concentration, red blood cell distribution width, platelet, leukocyte, creatinine, glutamate pyruvate transaminase, uric acid, cholesterol, C-reactive protein, potassium, sodium, blood urea nitrogen, triglycerides, total cholesterol, low density lipoprotein cholesterol, high-density lipoprotein cholesterol, amino-terminal pro-brain natriuretic peptide, high sensitivity troponin T, creatine kinase, glutamate oxaloacetate transaminase, gamma-glutamyl transferase, lipoprotein (a), glucose, HbA1c, ferritin)
- Magnetic resonance imaging (MRI); → stopped for the routine program in 3/2016
- Summarizing medical consultation
